# Drinking, Substance Use and the Operation of Motor Vehicles by Young Adolescents in Canada

**DOI:** 10.1371/journal.pone.0042807

**Published:** 2012-08-22

**Authors:** William Pickett, Colleen Davison, Michael Torunian, Steven McFaull, Patricia Walsh, Wendy Thompson

**Affiliations:** 1 Department of Emergency Medicine, Queen's University, Kingston, Ontario, Canada; 2 Clinical Research Centre, Kingston General Hospital, Kingston, Ontario, Canada; 3 Department of Community Health and Epidemiology, Queen's University, Kingston, Ontario, Canada; 4 Strategic Policy and Research Section, Division of Childhood and Adolescence, Public Health Agency of Canada, Ottawa, Ontario, Canada; 5 Injury Section, Health Surveillance and Epidemiology Division, Public Health Agency of Canada, Ottawa, Ontario, Canada; Centre for Addiction and Mental Health, Canada

## Abstract

**Background:**

Impaired driving is a recognized cause of major injury. Contemporary data are lacking on exposures to impaired driving behaviours and related injury among young adolescents, as well as inequities in these youth risk behaviours.

**Methods and Findings:**

Cycle 6 (2009/10) of the Health Behaviour in School-Aged Children survey involved 26,078 students enrolled in 436 Canadian schools. We profiled cross-sectionally the reported use of alcohol, marijuana, or other illicit drugs by on-road and off-road vehicle operators when young adolescents (mean age 13.3 (±1.6) years) were either driving or riding as a passenger. Comparisons were made across vulnerable subgroups. Multi-level logistic regression analyses were used to quantify the effects of the driving behaviours on risks for motor vehicle-related injury. Attributable risk fractions were also estimated. A total of 10% (±3%) of participants reported recent operation of an on-road or off-road motor vehicle after consuming alcohol, marijuana, or other illicit drugs, while 21% (±3%) reported riding as a passenger with a driver under the same conditions. Larger proportions of youth reporting these risk behaviours were males, and from older age groups, rural communities, and socio-economically disadvantaged populations. The behaviours were consistently associated with increased risks for motor vehicle-related injury at the individual level (RR 2.35; 95% CI: 1.54 to 3.58 for frequent vs. no exposure as a driver; RR 1.68; 95% CI: 1.20 to 2.36 for frequent vs. no exposure as a passenger) and at the population level (Attributable Risk Fraction: 7.1% for drivers; 14.0% for passengers). The study was limited mainly by its reliance on self-reported data.

**Conclusion:**

Impaired driving is an important health priority among young adolescents in Canada. Inequities in the involvement of younger adolescents in these risk behaviours suggest the need for targeted interventions for specific subgroups such as youth from rural communities, and among socially disadvantaged populations.

## Introduction

Within Canada, injury is the third leading cause of death in males and the fifth leading cause of death in females from the general population. [1] The economic burden caused by injury is second only to cardiovascular disease, most recently estimated to cost Canadians $8.7 billion annually in direct ($4.3 billion) and indirect ($4.4 billion) economic losses. [2] These facts, along with the recognition that injury control research is a relatively new discipline have led pediatric injury to be identified as a leading priority for health research and prevention. [3]

It is clear, at least among adults, that some populations are more vulnerable to injury than others. For example, there are distinct patterns of injury based on age, sex, geographic settings, racial groups and socio-economic status. Males are much more likely than females to be involved in motor vehicle crashes;[2] people from rural settings are involved in more collisions with subsequent injuries than in urban areas;[4] and Aboriginal peoples also experience disproportionately high risks for injury. [5]–[6] Young people are also especially vulnerable. In 2005, 720 children under the age of 20 (64% of whom were 15–19) were killed in Canada due to injury. [4] Person years of life lost attributable to injury in Canada in the 10–19 year old age group were conservatively estimated at 50,968 per year based upon 2007 mortality rates and life expectancy estimates. [7] An estimated 1,500 to 1,850 children under the age of 20 are hospitalized with major trauma each year. [8]–[9] When viewed collectively, injury is responsible for more deaths and hospitalizations than any other cause of ill health in the adolescent age group. What is not entirely clear is whether, like in the adult population, risks for injury and its adverse outcomes are differentially experienced by specific subgroups of young adolescents in Canada.

On-road and off-road motor vehicle-related crashes are among the most common causes of major injury affecting young Canadians. [4], [8]–[9] Substantial proportions of these injury events are caused by vehicle operators who are impaired by alcohol or other drugs. [10] Over the last decade, there have been increases in the number of alcohol and drug-related motor vehicle-related crashes in North America. [11]–[12] In 2009 in Canada, 2,575 people were killed in motor vehicle-related crashes involving pedestrians or occupants of vehicles, and in an estimated 976 (38%) of these events alcohol was determined to be a contributing factor. [13] Injury events involving off-road vehicles are an important component of these motor vehicle-related crashes. [12], [14] Ridership of off-road vehicles in North America is substantial, especially in rural settings when used both as a fundamental mode of transportation and as a common recreational pursuit. [15]–[16] Use of alcohol and other drugs by off-road vehicle operators are established risk factors for major injury to both drivers and their passengers. [17] Alcohol, cannabis and other drugs have direct individual and also possible combined effects that cause driver impairment. [18]–[19] In the United States, more than 500 children under the age of 16 are killed annually in alcohol or drug-related motor vehicle-related crashes. [20] Nearly half of these fatalities involve children killed as passengers in vehicles being operated by a driver impaired by alcohol or other drugs.

Our research group has a special interest in injury, its antecedent causes and its consequences in populations of young people. We are also interested in understanding more about inequities that exist in the risk behaviours and experiences of related injury among young adolescent populations. An inequity is an *avoidable and unfair difference* where judgments about fairness must be made within specific settings and societal contexts. [21] It is unfair, for example, that young people from specific types of communities experience higher risks for injury, or that a child who is born in poverty experiences elevated injury rates. Inequities often have root causes related to socio-cultural norms or aspects of systems, structures, institutions or routines that are important to understand and target for preventive interventions. Seldom have such factors been explored for youth injury in our country.

We were interested specifically in identifying inequities in risk behaviours between different socio-demographic groups of Canadian youth. This interest area has been fostered through our involvement in the conduct of a survey called *Health Behaviour in School-Aged Children (HBSC)*. [22] We used Cycle 6 of the Canadian HBSC to perform a national study in order to: (1) describe the overall prevalence of potential impaired driving behaviours among young people during the early adolescent years (average ages 11–15); ages that are typically below those required to obtain a drivers' license; (2) to describe variations in reports of such behaviours between population subgroups defined by socio-demographic factors, in order to identify potential inequities and sources of “unfair” patterns that exist among adolescent populations; (3) to estimate the impacts of these behaviours on risks for motor vehicle-related injury at both the individual and population levels. Through these analyses we provide the international public health community with basic evidence to support efforts aimed at addressing major injuries to young people. Such efforts include re-establishment of impaired driving as a priority for health promotion, the identification of vulnerable population groups for targeted intervention, and educational and policy-oriented solutions to the problem.

## Methods

### Sample

HBSC is a cross-national research study conducted in collaboration with the World Health Organization. [22] This study aims to increase understanding of health and its determinants in populations of young people. It involves written health surveys conducted with students in classroom settings, with a focus on the early adolescent years (ages 11–15; typically grades 6–10 in Canada). The national study protocol was approved by the General Research Ethics Board at Queen's University.

HBSC is administered every four years following a common international protocol. [22] Cycle 6 of the Canadian HBSC was conducted in 2009–10 and involved participants in 8 provinces and the 3 territories (all but Prince Edward Island and New Brunswick participated; over-samples were administered in certain provinces (British Columbia, Alberta, Saskatchewan and Newfoundland and Labrador) and a census of all young people attending school was attempted in the 3 northern territories (Yukon Territory, North West Territories, and Nunavut). The national sample was stratified by province/territory, type of school board (public vs. separate), urban-rural geographic status, school population size, and language of instruction (French vs. English) with standardized population weights generated to account for the oversampling and stratification criteria. Children from private schools, home school situations, Native reserves, street youth, incarcerated youth, and youth not providing informed consent (explicit or implicit, as per local school board customs) were excluded. Sampling was conducted with replacement; when schools that were approached were unable to participate, a school in the same or neighboring board with a similar demographic profile as per the stratification criteria was substituted. The survey was administered during the months of November 2009 through May 2010.

### Inclusions

Inclusion criteria were: (1) provision of informed consent by a parent or guardian of each child, using either an active or passive process as per local school board requirements and as approved by our institutional review board; (2) participation of the child in the 2009–10 Canadian HBSC; (3) valid responses to a questionnaire module describing risk factors for major injury including potential impaired driving behaviours; (4) provision of basic demographic information. The latter permitted the identification of groups for the proposed health inequity analysis. There were no specific exclusions beyond the initial sampling criteria. Response rates were 11/13 (84.6%) at the province/territorial level, 436/765 (57.0%) at the level of schools, and 26,078/33,868 (77.0%) at the individual student level. Decisions to employ explicit *vs.* implicit consent processes varied by school board; in jurisdictions that required explicit consent procedures, a 69.8% response rate was achieved; this increased to 81.2% in jurisdictions that required implicit consent procedures. Percentages of schools that used explicit consent varied by province, from a low of 21/58 (36.2%) of schools in British Columbia to a high of 9/13 (69.2%) of schools in Manitoba. Only implicit consent was used in the three Territories.

### Primary study variables

These included items describing potential exposures to potential impaired driving, socio-demographic factors (sex, age group, urban-rural geographic setting, country of birth and (if not) years resident in Canada, and socio-economic status), reports of motor vehicle-related injuries in the past 12 months, and other major risk factors for injury that could potentially confound the etiological relationships of interest.

#### Impaired driving module

A series of items were adapted froman existing survey instrument from Manitoba. For a 30 day period of recall, participants were asked “*how many times did you ride in a car or other vehicle (i.e., snowmobile, all- terrain vehicle, dirt bike) driven by someone who had been drinking alcohol, using marijuana or other illegal drugs*?”; then “*how many times did you drive a car or other vehicle (as above) when you had been drinking alcohol, using marijuana or other illegal drugs?*”. There were 3 response options for each item (*“never”, “1–3 times” (infrequent), “4 or more times” (frequent)).* Participants reporting these behaviours were considered to be potentially exposed to impaired driving behaviours. Field testing prior to the 2010 indicated that the items have high levels of face validity in that they were well and clearly understood by participating students. However, because it is impractical for researchers to systematically compare self-reports of such behaviours to direct observations in real life situations, these items have not been subjected to tests of validity in its truest sense. Further, as we were unable to determine whether the self-reported use of alcohol or other drugs was sufficient to cause impairment to legal levels from these items, we use the term “potential impaired driving” in this analysis.

#### Socio-demographic factors

These included participant sex (*male or female*), age group (*<13, 13 to 14, ≥15 years; corresponding with different periods of adolescent development*), years of residence in Canada (*born in Canada, 1 to 5 years, >5 years*), and socio-economic status measured by “how well off do you think your family is? (5 response options: “*very well off*” through “*not at all well off*”; later categorized into 3 categories). We also considered the size of the school community using divisions of urban-rural geographic status (*“Rural” {population <1000 persons}*, “*Small Centre*” *{1000 to 29,999 persons}*, *“Medium Centre”{30,000 to 99,999 persons} and “Large Urban Centre” {100,000 or more persons}*).

#### Motor Vehicle Injuries

Using a module adapted from US population health surveys, [23] participants were asked to report the occurrence of medically treated injuries. First, they were asked an initial screening question: “*During the past 12 months, how many times were you injured and had to be treated by a doctor or nurse?*” (“*I was not injured*”, “*1 time*”, “*2 times*”, “*3 times*”, “*4 times or more*”). Students reporting one or more injuries were asked supplemental questions about their most serious injury event. This included one item used to describe the activity leading to the injury, which included one response option “*riding/driving in a car or other motorized vehicle*”. The latter were defined as *motor vehicle-related injuries*.

### Analysis

We first described the study population demographically. Next, potential exposures to potential impaired driving behaviours were estimated within the full national sample, then within population subgroups defined by sex, age group, urban-rural geographic status, years resident in Canada, and socio-economic group. Prevalence estimates were weighted proportional to their respective standardized sampling fractions. Available sample sizes were sufficient to provide prevalence estimates that were ±2% when the entire sample was employed, and up to ±4% when sub-samples were used. We then conducted a series of multi-level logistic regression analyses using the SAS Procedure PROC GLIMMIX, with students nested within schools, to study the potential influence of impaired driving behaviours as possible determinants of motor vehicle-related injuries. We used a purposeful modeling strategy, with consideration of past evidence, theory, and model parsimony for the selection of covariates. Relative risks and associated 95% confidence intervals were estimated with inflation of standard errors used to account for the clustered nature of the sample. Population attributable risk (PAR) estimates were calculated to estimate proportions of motor vehicle-related injuries observed in the national population that were attributable to the potential impaired driving behaviours.

## Results

A total of 26,078 young people from 436 schools participated; of these 24,366 provided valid responses to key descriptive variables, and 23,212 provided full data required for the final regression analysis. The sample is described demographically in **Table 1** **.** It included children in their early adolescent and adolescent years that were concentrated in grades 6 to 10 within elementary, secondary and mixed grade school types. Varying economic, geographic and other demographic groups were well represented in order to permit meaningful subgroup analyses and to more fully examine inequities in health risk behaviours.

**Table 1:** Characteristics of student sample in the 2010 Canadian HBSC Survey.

**Characteristic**		
***Demographic***		
**Age**		
Mean (SD)	13.3	(1.6)
Range in years	9 to 19	
**Sex**		
Boys	12,815	(49.2)
Girls	13,254	(50.8)
**Grade level**		
<8	10,370	(39.8)
8 to 9	10,661	(40.9)
≥10	5,047	(19.4)
**Socio-economic status**		
Well off	13,998	(56.9)
Average	8,276	(33.6)
Not well off	2,339	(9.5)
**Urban-rural geographic status**		
Large urban population centre	7,030	(27.0)
Medium population centre	6,799	(26.1)
Small population centre	10,272	(39.4)
Rural	1,977	(7.6)
**Years in Canada**		
Born in Canada	18.466	(71.5)
≥5	6,143	(23.8)
1 to 5	1,212	(4.7)
***Impaired driving measures***		
**Rode in car or other vehicle in last 30 days with potentially impaired driver**		
None	19,174	(78.&)
1 to 3 times	2,326	(9.6)
4 or more times	2,866	(11.8)
**Drove a car or other vehicle in last 30 days while potentially impaired**		
None	21,887	(89.9)
1 to 3 times	1,440	(5.9)
4 or more times	1,030	(4.2)

Approximately 1 in 5 of the participants reported riding as a passenger in a car or other motor vehicle in the last 30 days with a driver who had been drinking, using marijuana or other illegal drugs, and 1 in 10 reported driving in a car or other motor vehicle while in such a situation (**Table 1**).

Within subgroups defined by demographics, the prevalence of both potential impaired driving behaviours was higher in males *vs.* females (p<0.001), and increased as the youth entered the older grade levels (p_trend_<0.001; **Table 2**). Youth from rural settings reported the highest levels of involvement in both behaviours (p_trend_<0.001). For *riding as a passenger*, strong and statistically significant increases in prevalence were evident across geographic categories but only among boys (p_trend_<0.001) and in the oldest (13 to 14, ≥15) age groups (p_trend_<0.001). For *driving a motor vehicle*, this urban-rural pattern of risk was evident in all population subgroups defined by age and sex (p_trend_<0.001).

**Table 2:** Percentage of young adolescents reporting any use of alcohol, marijuana or other illicit drugs by driver while in a car or other motor vehicle, 2010 Canadian HBSC by sex and age group.^1^

Risk Behaviour	Total population	By Sex		By Age Group		
		Boys	Girls	<13	13 to 14	≥15
	(n = 24,366)	(n = 11,713)	(n = 12,648)	(n = 8,270)	(n = 9,644)	(n = 6,452)
	Percentage of total or subgroup					
**Riding as a passenger**						
All respondents (total)	21.3	22.3	20.5	17.4	20.4	27.8
***By urban-rural geographic status***						
Large urban centre	20.5	20.0	20.9	17.0	20.2	24.3
Medium centre	19.7	21.2	18.3	17.1	18.5	26.1
Small centre	22.4	23.5	21.3	17.4	20.9	31.5
Rural	26.6	33.8	19.5	22.1	26.6	33.6
**Driving motor vehicle**						
All respondents (total)	10.1	12.8	7.7	6.9	10.1	14.3
***By urban-rural geographic status***						
Large urban centre	9.1	10.9	7.3	6.3	9.6	11.1
Medium centre	7.8	10.6	5.1	6.0	7.6	10/9
Small centre	11.8	14.6	9.4	7.2	11.5	18.6
Rural	15.1	22.5	7.8	13.2	13.7	20.6

1 all percentages accurate to within ±2%.

Inequities in the two behaviours were further described by geographic setting, years resident in Canada, and perceived socio-economic status (**Table 3**). Relative to those born in Canada, new immigrants (1 to 5 years resident in Canada) and other immigrants (>5 years) reported equivalent prevalence values for *riding as a passenger* (p = 0.21). New immigrants reported lower prevalence values for *driving a motor vehicle* in such a condition compared with the other two groups (p = 0.002), with slight variation by urban-rural geographic status. Those from “below average” socio-economic backgrounds also reported higher levels of engagement in both behaviours (p<0.001).

**Table 3:** Percentage of young adolescents reporting any use of alcohol, marijuana or other illicit drugs by driver while in a car or other motor vehicle, 2010 Canadian HBSC by years in Canada and socio-economic status.

Risk Behaviour	By Years in Canada			By Socio-economic Status		
	Born in Canada	>5 years	1 to 5 years	Above average	Average	Below Average
	(n = 17,368)	(n-5,674)	(n = 1,095)	(n = 13,426)	(n = 7,844)	(n = 2,196)
	Percentage of total or subgroup^1^					
**Riding as a passenger**						
All respondents (total)	21.4	21.6	19.3	19.3	22.3	29.8
***By urban-rural geographic status***						
Large urban centre	20.8	20.4	18.1	18.2	21.2	30.8
Medium centre	19.3	21.0	22.0	17.6	21.6	26.2
Small centre	22.5	22.2	20.2	20.8	22.7	30.6
Rural	25.7	31.1	19.4	22.8	30.0	32.4
**Driving motor vehicle**						
All respondents (total)	10.0	11.1	7.9	9.7	9.8	13.2
***By urban-rural geographic status***						
Large urban centre	8.9	10.3	7.0	7.8	9.9	12.4
Medium centre	7.5	9.2	6.7	7.2	7.9	9.8
Small centre	11.7	12.0	16.3	12.3	10.2	14.6
Rural	14.2	19.8	2.6	13.6	14.8	21.3

1 all percentages accurate to within ±3%, with the exception of by years in Canada 1 to 5 years which is ±4%.

Children who frequently engaged in either of the two potential impaired driving behaviours also reported modest and statistically significant increases in risk for motor vehicle-related injuries relative to those with no such reported behaviours (**Table 4**). These associations remained after adjustment for group differences by sex, age group, urban-rural geographic setting, years resident in Canada, and socio-economic status. **Table 5** further summarizes the potential impact of these exposures on risks for injury at the population level. An estimated 14.0% of the reported motor vehicle-related injuries were attributable to riding in a motor vehicle with a driver who had been drinking or using drugs, while 7.1% of motor vehicle-related injuries were attributable to driving a motor vehicle under such conditions.

**Table 4:** Results of multiple logistic regression analysis examining direct effects of driving behaviours on risks for motor vehicle injury; 2010 Canadian HBSC survey.

Use of alcohol or drugs while:	No.	% Injured	Crude Relative Risk^1^		Adjusted Relative Risk^1^^,^ 2	
			RR	(95% CI)	RR	(95% CI)
**Riding as a passenger**						
Never	18,637	0.9	1.00		1.00	
Infrequent	2,252	1.8	1.86	(1.32 to 2.64)	1.73	(1.21 to 2.48)
Frequent	2,736	1.7	1.72	(1.24 to 2.40)	1.68	(1.20 to 2.36)
**Driving motor vehicle**						
Never	21,282	1.0	1.00		1.00	
Infrequent	1,372	1.5	1.35	(0.84 to 2.15)	1.29	(0.81 to 2.06)
Frequent	971	2.9	2.56	(1.70 to 3.84)	2.35	(1.54 to 3.58)

1 Estimated using multi-level procedures; students nested within schools, and SAS PROC GLIMMIX Procedure.

2 Model was adjusted for the following factors: sex, age group, socio-economic status, urban-rural geographic status, years in Canada.

**Table 5:** Population Attributable Risk Fraction Estimates: 2010 Canadian HBSC Survey.

Population	% Exposed	Adjusted RR^1^	PAR %
**Riding as a passenger**			
Infrequent	11.8	1.73	7.9
Frequent	9.6	1.68	6.1
Total	21.4		14.0
**Driving motor vehicle**			
Infrequent	5.9	1.29	1.7
Frequent	4.2	2.35	5.4
Total	10.1		7.1

1 Model was adjusted for the following factors: sex, age group, socio-economic status, urban-rural geographic status, years in Canada.

## Discussion

This national study has a number of important findings. First, it reports that substantial proportions of young people in their early adolescent years report recent driving of a car or other off-road motor vehicle after consuming alcohol, marijuana or other illicit drugs, or riding as a passenger in a car or off-road motor vehicle with a driver who has consumed alcohol, marijuana or other illicit drugs. Second, it demonstrates that clear inequities exist in the engagement in these health risk behaviours, with the highest proportions reported by youth from rural communities, males, older age groups, and socio-economically disadvantaged populations. Finally, it shows that these behaviours are not innocuous in terms of their potential health consequences. Reports of potential impaired driving were consistently associated with increased risks for motor vehicle injuries at the level of the individual student and at the population level.

Substantial portions of young adolescent Canadians are exposed to potential impaired driving behaviours. Given the magnitude of the traumatic motor vehicle-related injury problem in North America, [11]–[12] and the fact that approximately one-third of fatal crashes can be attributed to impaired driving, especially in populations of youth, [13]–[14] this is quite significant. Despite being established as a major risk factor for traumatic injury, and being the focus of a myriad of educational and regulatory interventions, [24]–[25] such behaviours clearly persist. Our study varied from most efforts used to quantify such behaviours because of our specific focus on young adolescents, and our use of an inclusive definition of vehicle use that considered both on-road vehicles such as cars, and off-road vehicles such as snowmobiles, all-terrain vehicles, and dirt bikes. We studied populations of young adolescents who, with few exceptions, had not reached the age required to obtain a driver's license in their province or territory. Our focus on driving contexts that extend beyond typical on-road situations is also novel to the public health literature. Taken together, our findings suggest that impaired driving is a public health problem that extends to populations of children in their early teenage years. This problem also extends beyond the typical on-road drinking and driving scenarios, to ones that include use of alcohol and other drugs on a variety of off-road vehicle types.

A major purpose of our study was to investigate inequities in exposure to potential impaired driving behaviours among young people in Canada and related injury. Through this analysis, we provided basic evidence about variations in high-risk behaviours. Increases in exposure to potential impaired driving among boys and students in the older age groups were expected given well known gender differentials in risk-taking and substance use, [26] as well as the increase in motor vehicle use anticipated with reaching the age of possible drivers license acquisition in Canada. The consistency of observed increases in risk reported by young people from rural communities was of greater interest as a potentially modifiable health inequity. Such youth populations are known to suffer considerable disadvantages when it comes to their health, whether measured in terms of standard indicators of mortality and morbidity, as well as their underlying causes. [4], [27]–[28] Our analysis extends this finding for rural settings to injury-related health behaviours.

Several explanations exist for the higher reported exposures to potential impaired driving behaviours among young people from rural communities. Because most members of our study population were not old enough to hold a driver's license, a portion of these increases are attributable to youth riding as a passenger in a motor vehicle or while using off-road vehicles. All-terrain vehicles, snowmobiles and dirt bikes can be operated legally by minors on private property, and their use is more common in rural settings. Second, differences in the built environment and the policies that govern it may exist between rural and urban areas. In urban areas, there are typically formal options for travel for young people including public transit and taxis. Such options do not exist to the same extent in more rural communities, leading to greater reliance on private transportation and informal arrangements for travel, with accompanying risks for impaired driving. Differences in cultural norms surrounding overt risk-taking may also exist with a coincident greater tolerance for such risk behaviours in some cultures. Drinking and driving behaviours exhibited by parents are highly correlated with increased risks for such behaviours in new drivers. [29] Speculatively, this phenomenon might contribute to higher reported exposures to potential impaired driving behaviours in rural areas should such communities be home to a drinking and driving culture that crosses generations.

Our analysis provides evidence for the existence of additional health inequities. To illustrate, high levels of engagement in potential impaired driving behaviours were reported by young people from socio-economically disadvantaged populations in all urban-rural community settings. These social variations are obviously complex and multi-factorial in terms of their origins. Population health theory, and most recently the report on the WHO Commission on the Social Determinants of Health, [30] suggests that determinants of health involve both individual factors as well as characteristics of the social and physical settings in which these factors are manifested. It is clear that at least part of the observed inequities in injury-related risks are attributable to differences in overt risk behaviours, but that these behaviors are likely affected, if not caused by, varying social norms within youth cultures as well as aspects of their social and built environments. We feel our findings confirm those of others [30] indicating that health is determined in part by individual behaviour, but that features of social and physical environments may be at the root of observed unfair differences in health risks and adverse health outcomes. These differences require further and deeper exploration.

Strengths and limitations of this study warrant comment. Major strengths include the novelty of this analysis with respect to its focus onpotential impaired driving in young adolescents, its national scope, and its focus on injury-related health experiences of an important yet understudied group. Foundational evidence gained from our analyses provides a first step for the planning of effective countermeasures for adolescent populations. Our confirmation of inequities between several vulnerable population subgroups too points to the complex etiology of injury-related health disparities. This is of importance to public health at the population level.

The study also has several limitations. The HBSC survey is necessarily reliant on the provision of accurate information from children obtained via self-report. While considerable efforts are made to use established questionnaire items that have been subject to checks for reliability, [22] some misclassification is inevitable. Second, participation in the HBSC was voluntary and the response rate was 59% at the school level and 77% at the individual student level, with variation in response rates according to whether implicit or explicit consent was used. It is possible that non-respondents had differential experiences with the impaired driving and associated injury measures. For example, requirements for explicit consent very likely decreased response among groups of young people who were most vulnerable to the potential impaired driving behaviours leading to underestimates of prevalence and risk. Such patterns of non-response are unlikely to affect relations between the impaired driving behaviours and injury save for a loss of statistical power expected due to lower numbers of exposed students being available for analysis.

Our use of composite measures for potential impaired driving behaviour represents a third limitation. It was not possible to establish the exact driving contexts in which these potential impaired driving behaviours occurred. We were unable to distinguish, for example, the use of alcohol from other drugs prior to driving or riding and subsequently quantify any differences in the effects of specific types of substance use on risks for injury. For example, when alcohol and cannabis are taken together, some argue that it is the alcohol that is consumed that is solely responsible for any risk increase [18], [31] while others conclude that use of either substance, alone or in combination, contributes to such risks [19], [32]. We were unable to develop analyses to assist in resolving these debates. Fourth, because of our use of a composite measure for motor vehicle use, we were unable to distinguish the exact types of vehicles being operated and the contexts in which potential impaired driving behaviour occurred. As study participants were below the legal age for driver's license acquisition in most provinces and territories, almost all reported vehicle operation likely took place in off-road situations. Knowledge of the context of these specific risk-taking behaviours would assist in prevention efforts. In future cycles of the Canadian HBSC, the questionnaire items will be modified to provide this level of specificity. Finally, these are cross-sectional analyses and therefore caution is warranted in the causal interpretation of the observed relationships in the absence of longitudinal confirmation.

Our findings have implications for adolescent health policy. Ongoing surveillance of potential impaired driving behaviours in populations of young people, including those below the typical ages for driver's license acquisition, is of inherent value to policy development. Not only do our surveillance efforts quantify the magnitude of these problem behaviours and establish the need for public health interventions, they also point to vulnerable groups within the Canadian population and the possible combination of factors affecting their risk for, and experience of, impaired driving related injury. Surveillance findings also help to target prevention efforts. Surveillance also provides one potential basis for measuring the impacts of ongoing public health programs. Our findings of higher reported levels of exposure to potential impaired driving in rural communities and socio-economically disadvantaged groups call for innovative solutions specific to local contexts. Prevention strategies that are known to be effective include innovations in public and private transportation options for young people where possible, increased enforcement of impaired driving laws for both on-road and off-road vehicles, establishment of behavioural contracts between parents and teens, as well as targeted public education initiatives. [33]–[35] Finally, there is a need for more policy-oriented research, both etiological and intervention, that is centered upon the root causes of impaired driving in Canada and its inequities. The latter may include furthering understanding of the etiology of problem drinking and substance use behaviours in young people, the social conditions that determine such behaviours, and sensible points of intervention within specific community settings.

In summary, this Canadian analysis examined potential impaired driving behaviours among populations of young adolescents using contemporary health survey data. Such behaviours were common in the overall study population, and also were related to the occurrence of higher risks for motor vehicle-related injury. Reported inequities in the involvement of young people in these risk behaviours were identified, and suggest the need for targeted interventions within rural communities, and among socially disadvantaged populations of Canadian youth. These behaviours and inequitable high-risk groups clearly remain as priorities for public health efforts within adolescent populations in our country.
